# The Book World of Medicine and Science

**Published:** 1897-05-22

**Authors:** 


### The Book World of Medicine and Science.

Royal Infirmary Cliniques. By Alex. James, M.D., F.R.C.S.E. Post 8vo., pp. 166. Illustrated. (Edinburgh: Oliver and Boyd. 1896.)

This small volume, containing sixteen clinical lectures by a teacher of experience, belongs to a class of medical literature which is especially valuable to the advanced student of medicine and to young practitioners. When a fair knowledge of systematic medicine has been acquired, it is by hearing such lectures as these, or by reading them, that practitioners and students are enabled to appreciate the lessons of individual cases as they are viewed from the standpoint of a cultured physician who, by long practice in teaching, has acquired those methods of scientific precision which are invaluable at the bedside, and which distinguish the clinician from the bookworm.

The Private Sanatoria for Consumptives and the Treatment Adopted Within Them. By Dr. Von Jaruntowsky. Translated by E. Clifford Beale, M.A., M.B. Cantab., F.R.C.P. Pp. 48, large 8vo. (London: Rebman Publishing Co. 1896.)

Dr. Beale has brought before English readers much information in a brief form which will be useful to physicians who wish to compare the relative advantages of different sanatoria, as far as written information can go, and to note the different methods adopted in treatment. Not the least valuable is the introduction on pulmonary gymnastics, dietetic treatment, &c.

On the Plans of Modern Asylums for the Insane Poor. By John Sibbald, M.D., Commissioner in Lunacy for Scotland. (Printed by Jas. Turner. 1897. pp. 33, 8vo.)

This is a reprint of a paper by the author designed to give information to the lunacy authorities of the Edinburgh district, and deals in a concise way with some aspects of asylum construction. The author emphasises the view—ever to be prominently borne in mind—of regarding the asylum not only as a medical institution, but also as a home of residence—often for long periods of time—for the patients; and in accordance with these views he advocates the planning, laying out of the buildings, in such a way as to suggest most strongly the appearance of a number of private houses or cottages, with gardens, &c., of which the asylum at Alt Scherbitz is such an excellent type. Some asylums, however, especially the older ones, with their prison-like towers, and other arrangements more strongly suggestive of the prison than of the asylum, are agreeable neither to the patient nor to the world at large, and convey an impression which should never even be hinted at. Great credit is given to Connolly for his pioneer efforts in promoting the reforms of asylum construction, in connection with the above idea. Doors with handles to turn instead of the "obnoxious key" are advocated as better in the corridors and rooms, though here obvious difficulties are present which make it impossible in many asylums. The author is not an admirer of the huge asylums, like those of London, of the present date; smaller ones for about 600 patients are probably the best. The system of pavilions—separate blocks, each with day-rooms, dormitories, and other accessories, and each suitable for a special class of patients and complete in itself—is the best hygienically, and most effective in developing the feeling of responsibility to the minor officers in charge. The separate blocks, of course, are connected with one another and with the central (medical and administrative) block by covered corridors. Claybury and Gartloch are good modern instances. One section or block should be devoted to the more acute and curable cases; this should be more fully and carefully officered, and fitted with all the newest therapeutic appliances. The author concludes by expressing his view that the best type of asylum is the scattered blocks and cottage or village type—like that at Alt Scherbitz—a type steadily growing in favour both in Germany and America. At home several of our private asylums approach this type; though apparently the same scheme would be hardly practicable for pauper asylums on the ground of cost. Yet it must be the ideal type, "for it carries further than any other type the idea of making asylum arrangements similar to those of ordinary life, and every development in asylum construction which has stood the test of experience has been made in this direction." Four plans of modern asylums are given. The book is to be highly commended.

Mediterranean Winter Resorts. By Eustace A. Reynolds-Ball. (London: Messrs. Kegan Paul.)

Mr. Reynolds-Ball's excellent little book has now passed into its third edition. We are not surprised to hear this, for his work embraces all necessary information on the subject, and such information is conveyed in a pleasant, chatty manner. The present edition is "considerably enlarged, and in great part re-written, several new and important features having been introduced," he tells us in the preface. The work is described as a practical handbook to the principal health and pleasure resorts on the shores of the Mediterranean; but Mr. Reynolds-Ball does not confine himself alone to this locality, but gives detailed information of those newer winter quarters, such as Biskra, Luxor, and Sicily, &c. Then, what is an invaluable aid to persons who propose wintering abroad, all sorts of useful hints are given as to various routes, cost of living, hotel expenses, &c. In fact, all and every information which could be required on the subject is to be found in Mr. Reynolds-Ball's work.

Black's Guide to Bournemouth and the New Forest. Edited by A. Hope Moncrieff. Illustrated with maps. 8vo., pp. 71. (London: Adam and Charles Black. 1896.)

This is a very handy, complete, and reliable guide to Bournemouth and its neighbourhood, and contains all the information which could be desired in a local guide in a well-arranged and very readable form.

